# Association of dynamic change of triglyceride-glucose index during hospital stay with all-cause mortality in critically ill patients: a retrospective cohort study from MIMIC IV2.0

**DOI:** 10.1186/s12933-023-01874-9

**Published:** 2023-06-17

**Authors:** Long Cheng, Feng Zhang, Wenjing Xue, Peng Yu, Xiaoyan Wang, Hairong Wang, Jun Wang, Tianyang Hu, Hui Gong, Li Lin

**Affiliations:** 1 Department of Cardiology, Jinshan Hospital of Fudan University, Shanghai, China; 2 Department of Urology, Shanghai Punan Hospital of Pudong New District, Punan Branch of Renji Hospital, No. 279 Linyi Road, Shanghai, 200000 China; 3 Institute of Cardiovascular Diseases, Ruijin Hospital, Shanghai Jiao Tong University School of Medicine, Shanghai, People’s Republic of China; 4 Department of Cardiovascular Medicine, Shanghai Pudong New Area Gongli Hospital, Shanghai, 200000 People’s Republic of China; 5 Department of Neurology, First People’s Hospital of Yancheng, Yulong Western Road, Yancheng, 224006 Jiangsu China; 6 Precision Medicine Center, The Second Affiliated Hospital of Chongqing Medical University, Chongqing, China; 7 Department of Cardiovascular Medicine,, Shanghai East Hospital, Tongji University School of Medicine, Jimo Road 150, Shanghai, 200120 China

**Keywords:** Triglyceride-glucose index, Intensive care unit, All-cause mortality, MIMIC-IV database

## Abstract

**Background:**

Biomarker of insulin resistance, namely triglyceride-glucose index, is potentially useful in identifying critically ill patients at high risk of hospital death. However, the TyG index might have variations over time during ICU stay. Hence, the purpose of the current research was to verify the associations between the dynamic change of the TyG index during the hospital stay and all-cause mortality.

**Methods:**

The present retrospective cohort study was conducted using the Medical Information Mart for Intensive Care IV 2.0 (MIMIC-IV) critical care dataset, which included data from 8835 patients with 13,674 TyG measurements. The primary endpoint was 1-year all-cause mortality. Secondary outcomes included in-hospital all-cause mortality, the need for mechanical ventilation during hospitalization, length of stay in the hospital. Cumulative curves were calculated using the Kaplan–Meier method. Propensity score matching was performed to reduce any potential baseline bias. Restricted cubic spline analysis was also employed to assess any potential non-linear associations. Cox proportional hazards analyses were performed to examine the association between the dynamic change of TyG index and mortality.

**Results:**

The follow-up period identified a total of 3010 all-cause deaths (35.87%), of which 2477 (29.52%) occurred within the first year. The cumulative incidence of all-cause death increased with a higher quartile of the TyGVR, while there were no differences in the TyG index. Restricted cubic spline analysis revealed a nearly linear association between TyGVR and the risk of in-hospital all-cause mortality (P for non-linear = 0.449, P for overall = 0.004) as well as 1-year all-cause mortality (P for non-linear = 0.909, P for overall = 0.019). The area under the curve of all-cause mortality by various conventional severity of illness scores significantly improved with the addition of the TyG index and TyGVR. The results were basically consistent in subgroup analysis.

**Conclusions:**

Dynamic change of TyG during hospital stay is associated with in-hospital and 1-year all-cause mortality, and may be superior to the effect of baseline TyG index.

**Supplementary Information:**

The online version contains supplementary material available at 10.1186/s12933-023-01874-9.

## Introduction

The utilization and costs associated with critical care medicine beds have been consistently increasing in the United States over the past three decades [1, 2]. This is a significant global public health concern due to the high mortality and morbidity rates, as well as the substantial economic burden [3–5]. Critical illness encompasses a diverse range of pathophysiological processes that can occur in severely ill or injured individuals, resulting in various functional defects, cellular dysfunctions, and organ impairments [6–8]. To determine disease severity and predict mortality in critically ill patients, several scoring systems that combine clinical features and biomarkers reflecting pre-existing health have been developed, such as Acute Physiology and Chronic Health Evaluation (APACHE) scores, Simplified Acute Physiology Scores II (SAPS II) et al. [9, 10]. While these systems have been validated across various settings and medical conditions, a limitation is their reliance on the worst physiologic or laboratory parameter collected within 24 h of (intensive care unit) ICU admission [11]. Without this information, these scores cannot be utilized. Therefore, it is necessary to explore new biomarkers in critically ill patients to identify more accurate predictors of disease severity and mortality.

Insulin resistance (IR) is a prevalent condition in critically ill patients and is considered a marker of systemic inflammatory response and metabolic disorders [12]. Previous studies have shown that insulin sensitivity is reduced by up to 70% in critically ill patients [6, 13], and this reduction is associated with illness severity, rather than admission diagnosis [6]. Although initially considered an adaptive response to trauma or sepsis, IR has been linked to significant morbidities in intensive care. Furthermore, changes in insulin resistance could indicate a more pronounced stress response inflammation, immune disorder [14, 15], which are not captured by existing severity scores. Therefore, it is crucial to continuously monitor and quantify IR in critically ill patients to better understand its impact on patient outcomes.

Triglyceride glucose (TyG) index, calculated from fasting triglyceride (TG) and fasting blood glucose (FBG), is a novel marker that has been well-recognized as a simple and reliable surrogate of IR. Recent studies have found that the TyG index is strongly associated with increased all-cause mortality in critically ill patients [16]. However, these studies only focused on the initial TyG value within the first 24 h of ICU admission and did not consider the dynamic changes in this marker over time. As insulin sensitivity in critically ill patients can change over time, and IR is closely linked to stress response inflammation and immune response to critical illness and its severity [17, 18], it is possible that the TyG variability ratio (TyGVR) during hospital stay may be a better marker for adverse long-term prognosis and could be used for early risk stratification in critically ill patients. However, no relevant studies have been conducted to explore this hypothesis. To address this gap, we conducted this study to examine the relationship between TyGVR and adverse prognosis and determine the superiority of prognostic prediction and stratification.

## Methods

The data presented in this study was extracted from Medical Information Mart for Intensive Care IV (MIMIC-IV version 2.0), a comprehensive collection of health-related data pertaining to 76,943 ICU stays for 53,150 unique patients (as computed by the authors) who received critical care at the Beth Israel Deaconess Medical Center between 2008 and 2019. It is noteworthy that none of the patients included in the study had contracted COVID-19. In order to preserve patient privacy, all personally identifiable information has been rendered anonymous. Given that all patient records in the MIMIC-IV database were fully de-identified, the requirement for individual patient consent was deemed unnecessary by the institutional review board of the Beth Israel Deaconess Medical Center.

### Cohort selection

**Fig.1:**
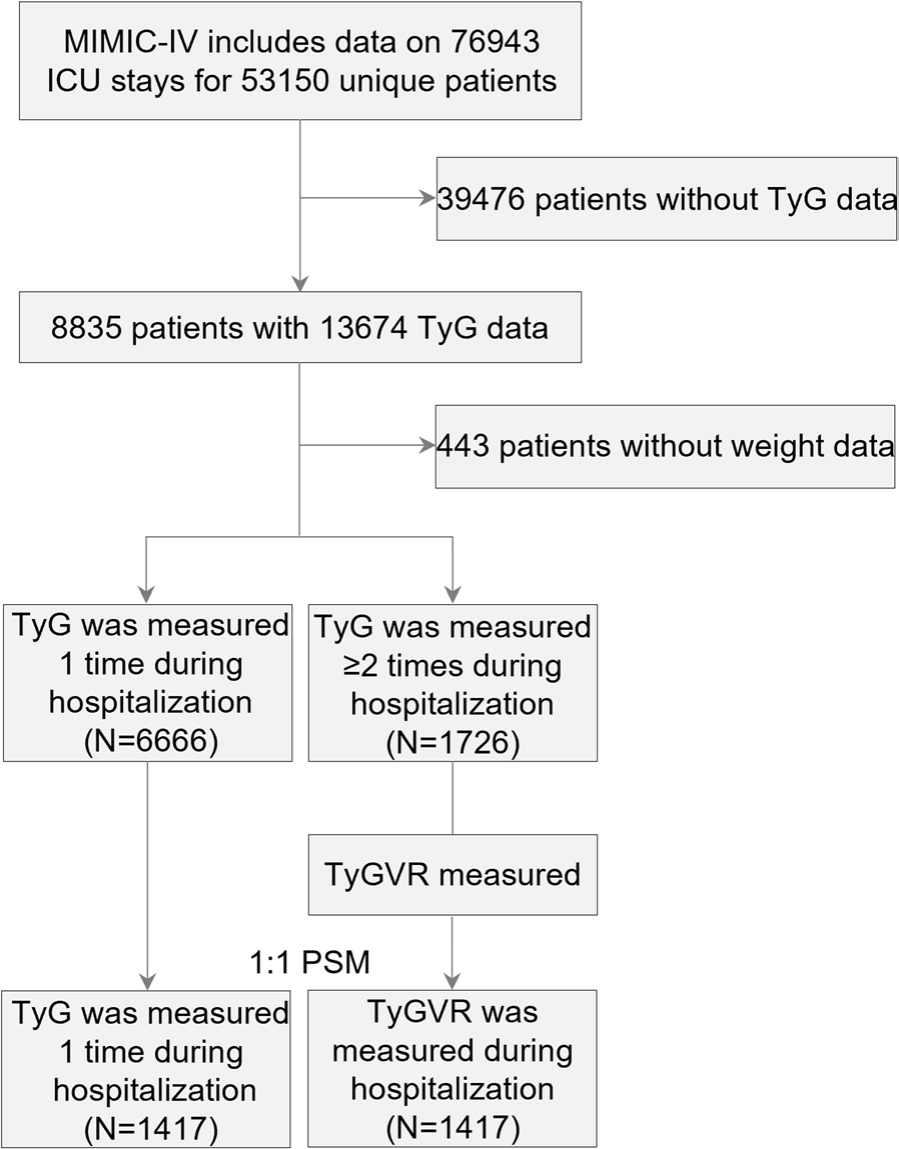
Flowchart of study participants. TyG index triglyceride glucose index, TyGVR triglyceride glucose index variability ratio

Selection criteria, as well as the number of patients, were excluded at each step, were shown in Fig. 1. The initial cohort was identified by the vital sign data of patients (aged ≥ 18 years) admitted to the ICU for the first time in the dataset. Of the 53,150 unique patients, 39,476 were excluded due to a lack of documented TyG data, while an additional 443 patients were excluded due to missing weight data. Ultimately, a total of 8392 patients were included in the final study cohort, which was divided into two groups based on the times of measurement of TyG index during hospitalization. Furthermore, baseline characteristics were presented based on the quartiles of the first day of ICU stay TyG index.

### Data extraction and definitions

All baseline characteristics, with the exception of TyG, were extracted within the first 24 h of ICU admission. The study data, which comprised patient demographics, comorbidities, survival outcomes, severity score, laboratory parameters, medication, and other relevant variables, were extracted by author L.C, who completed an online training course from the National Institutes of Health (with certification number 9046642) and obtained permission to access the MIMIC-IV database. The TyG index was determined using the formula ln (fasting triglycerides [mg/dL] × fasting glucose [mg/dL]/2) [16, 19]. TyGVR was calculated as follows:

$${\text{TyGVR}} = \, ({\text{TyG average }}{-}{\text{TyG baseline}})/ \, ({\text{TyG baseline}})$$

TyG baseline was calculated using the first recorded fasting blood glucose and triglycerides after ICU admission. TyG average was defined as the average value of fasting blood glucose and triglycerides recorded on multiple occasions, excluding the first time. All comorbidities were identified based on ICD-9 or ICD-10 codes. Sepsis was defined according to The Third International Consensus Definitions for Sepsis and Septic Shock [20]. Data extraction was performed using pgAdmin4 PostgreSQL 9.6. All human studies were approved by the appropriate ethics committee and were conducted in accordance with the ethical standards outlined in the 1964 Declaration of Helsinki and its subsequent amendments. This study adhered to the Strengthening the Reporting of Observational Studies in Epidemiology (STROBE) guidelines for observational studies.

### Outcomes

The primary outcome was 1-year all-cause mortality. Secondary outcomes included in-hospital all-cause mortality, the need for mechanical ventilation during hospitalization, length of stay (LOS) in ICU and hospital.

### Statistical analysis

For the baseline characteristics, data were presented as the mean with standard deviation (SD) or median with interquartile range (IQR) for continuous variables. The mean of continuous variables was compared using Student’s t-test or the Mann–Whitney U test. Categorical variables were expressed as quantity and frequency and were tested using Pearson chi-square test or Fisher’s exact test. Continuous variables with missing data exceeding 2% were excluded from the analysis to ensure the validity and reliability of the study findings. In order to minimize potential bias between the two study groups, propensity score matching (PSM) without replacement was performed using a caliper width of 0.2 of the pooled standard deviation of the logit of the propensity score. This matching was performed at a 1:1 ratio based on baseline factors to ensure comparability between the groups. An absolute standardized difference (ASD) < 20% for the measured covariate suggests an appropriate balance between groups. The baseline characteristics of the original and matched cohorts were listed separately. To explore the association between TyG index and TyGVR with the primary outcome of interest, the cumulative incidences of 1-year all-cause mortality were estimated using Kaplan-Meier curves and detected by the Log-Rank test. Potential nonlinear for the levels of TyG index and TyGVR with in-hospital and 1-year all-cause mortality measured by restricted cubic spline. Restricted cubic spline was used to assess potential nonlinear relationships between the levels of TyG index and TyGVR with in-hospital and 1-year all-cause mortality, with four knots located at the 5th, 35th, 65th, and 95th percentiles as per Harrell's recommendations [21, 22]. The presence of nonlinearity was assessed using the Wald test. Additionally, subgroup analyses were also performed to explore potential effect modification by variables such as sex, age, race and ethnicity, diabetes, obesity, hypertension, congestive heart failure, respiratory failure, cerebrovascular disease, anemia, chronic renal disease, liver disease, and sepsis 3.0. The interactions between TyG index and TyGVR with each of the above variables were tested, and findings were reported using hazard ratios (HR) or odds ratios (OR) with corresponding 95% confidence intervals (CI). Univariate linear regression models were used to estimate the association of the TyG index and TyGVR with length of ICU stay and length of hospital stay. The predictive value of TyG index and TyGVR, in conjunction with traditional severity of illness scores, was evaluated for in-hospital or 1-year all-cause mortality using receiver operating characteristic (ROC) and area under curves (AUC) analysis.These results were calculated from the matched patients.

All analyses were performed using STATA MP Version 16.0 (Stata-Corp, College Station, TX), and 2-side P < 0.05 was considered statistically significant.

## Results

### Cohort characteristics

As shown in Table 1, a total of 8392 eligible participants stratified by quartiles of TyG index were included in this study. The mean age was 64.44 ± 16.39 years, 57.21% were men and the average TyG index was 9.02 ± 0.81. During the follow-up period, 3010 (35.87%) all-cause deaths were identified, of which 2477 (29.52%) occurred within the first year. Compared with the Q1 group, patients in the other groups were more likely to be younger, male, higher prevalence of obesity, diabetes mellitus, respiratory failure, anemia, chronic renal disease, and sepsis3.0. Patients with higher TyG index tend to take hypoglycemic, vasopressin agents and had a higher level of white blood cell (WBC), serum were observed, blood urea nitrogen, fasting blood glucose, and triglyceride. Furthermore, in the higher TyG index group, longer LOS, ICU-LOS, and a higher proportion of invasive ventilation were observed.

**Table 1:** Baseline characteristics of the critically ill patients stratified by the TyG index quartiles

Categories	Overall (N = 8392)	Q1 (N = 2099)	Q2 (N = 2098)	Q3 (N = 2099)	Q4 (N = 2096)	*P-*value
TyG index, mean (SD)	9.02(0.81)	8.14(0.30)	8.71(0.12)	9.17(0.14)	10.09(0.67)	< 0.001
Demographic						
Age, years, mean (SD)	64.44(16.39)	67.54(17.09)	66.65(15.97)	64.25(15.76)	59.33(15.47)	< 0.001
Male, n (%)	4801(57.21)	1118(53.26)	1187(56.58)	1198(57.07)	1298(61.93)	< 0.001
Emergency, n (%)	6700(79.84)	1632(77.75)	1658(79.03)	1668(79.47)	1742(83.11)	< 0.001
Ethnicity, n (%)						< 0.001
White	5340(63.63)	1377(65.60)	1376(65.59)	1330(63.36)	1257(59.97)	
Black	762(9.08)	231(11.01)	186(8.87)	161(7.67)	184(8.78)	
Asian	232(2.76)	54(2.57)	50(2.38)	57(2.72)	71(3.39)	
Hispanic/Latino	270(3.22)	54(2.57)	48(2.29)	78(3.72)	90(4.29)	
Other	402(4.79)	94(4.48)	89(4.24)	108(5.15)	111(5.30)	
Unknown	1386(16.52)	289(13.77)	349(16.63)	365(17.39)	383(18.27)	
Obesity, n (%)	883(10.52)	110(5.24)	167(7.96)	246(11.72)	360(17.18)	< 0.001
Weight, Kg, mean (SD)	83.27(24.93)	76.78(23.11)	80.50(22.89)	84.40(24.58)	91.54(26.58)	< 0.001
Vital signs						
SBP, mmHg, mean (SD)	122.17(18.23)	122.35(18.47)	122.85(18.76)	122.27(18.00)	121.19(17.65)	0.029
DBP, mmHg, mean (SD)	65.97(12.01)	66.03(12.17)	65.97(11.84)	66.00(11.82)	65.88(12.19)	0.334
Respiratory rate, bmp, mean (SD)	19.69(4.02)	18.90(3.52)	19.39(3.73)	19.91(4.07)	20.57(4.49)	< 0.001
HR, bmp, mean (SD)	85.59(16.95)	82.37(16.15)	83.84(16.27)	86.32(16.60)	89.84(17.80)	< 0.001
Comorbidities						
Diabetes mellitus, n (%)	2448(29.17)	321(15.29)	437(20.83)	690(32.87)	1000(47.71)	< 0.001
Hypertension, n (%)	4570(54.46)	1123(53.50)	1179(56.20)	1141(54.36)	1127(53.77)	0.291
MI, n (%)	1807(21.53)	393(18.72)	473(22.55)	477(22.73)	464(22.14)	0.004
Respiratory failure, n (%)	2541(30.28)	416(19.82)	552(26.31)	690(32.87)	883(42.13)	< 0.001
CHF, n (%)	2152(25.64)	568(27.06)	536(25.55)	547(26.06)	501(23.90)	0.124
Cerebrovascular disease, n (%)	2930(34.91)	856(40.78)	847(40.37)	714(34.02)	513(24.48)	< 0.001
Anemia, n (%)	1628(19.40)	353(16.82)	397(18.92)	400(19.06)	478(22.81)	< 0.001
COPD, n (%)	1825(21.75)	418(19.91)	465(22.16)	452(21.53)	490(23.38)	0.052
Chronic renal disease, n (%)	1453(17.31)	328(15.63)	341(16.25)	362(17.25)	422(20.13)	0.001
Rheumatic disease, n (%)	286(3.41)	75(3.57)	76(3.62)	69(3.29)	66(3.15)	0.803
Peripheral vascular disease, n (%)	812(9.68)	198(9.43)	224(10.68)	195(9.29)	195(9.30)	0.366
Liver disease, n (%)	1260(15.01)	336(16.01)	249(11.87)	316(15.05)	359(17.13)	< 0.001
Dementia, n (%)	293(3.49)	113(5.38)	74(3.54)	66(3.14)	40(1.91)	< 0.001
Sepsis3.0^a^, n (%)	4311(51.37)	884(42.12)	952(45.38)	1144(54.50)	1331(63.50)	< 0.001
Laboratory parameters						
WBC, K/µL, mean (SD)	11.01(6.78)	9.84(4.74)	10.80(9.07)	11.42(5.22)	11.98(7.03)	< 0.001
RBC, m/µL, mean (SD)	3.58(0.70)	3.64(0.70)	3.65(0.69)	3.58(0.70)	3.46(0.70)	0.949
Platelet, K/µL, mean (SD)	229.95(107.16)	215.97(98.44)	230.97(104.20)	239.78(112.24)	233.08(111.81)	< 0.001
Hemoglobin, g/dL, mean (SD)	10.72(2.03)	10.92(2.00)	10.93(2.04)	10.70(2.03)	10.33(2.00)	0.673
Serum Sodium, mEq/L, mean (SD)	139.17(3.80)	138.81(4.02)	139.41(3.55)	139.31(3.74)	139.13(3.87)	< 0.001
Serum Potassium, mEq/L, mean (SD)	4.09(0.35)	4.07(0.35)	4.07(0.34)	4.08(0.34)	4.12(0.38)	< 0.001
Serum Calcium, mg/dL, mean (SD)	8.55(0.59)	8.60(0.57)	8.60(0.57)	8.53(0.59)	8.48(0.62)	0.004
Scr, mg/dL, mean (SD)	1.30(1.18)	1.16(0.99)	1.20(1.09)	1.30(1.19)	1.56(1.37)	< 0.001
Blood urea nitrogen, mg/dL, mean (SD)	25.57(17.77)	22.57(15.02)	23.73(15.64)	26.16(18.11)	29.82(20.84)	< 0.001
Glucose, mg/dL, mean (SD)	139.33(69.86)	105.45(25.33)	120.52(29.83)	141.53(48.05)	189.87(107.88)	< 0.001
TG, mmol/L, mean (SD)	168.82(212.57)	74.17(32.26)	110.94(42.13)	153.16(55.37)	337.24(366.25)	< 0.001
Medical history						
Antihypertensive agents, n (%)	6901(82.23)	1631(77.70)	1709(81.46)	1799(85.71)	1762(84.06)	< 0.001
Hypoglycemic agents, n (%)	5947(70.87)	1283(61.12)	1375(65.54)	1549(73.80)	1740(83.02)	< 0.001
Lipid-lowering agents, n (%)	4333(51.63)	1075(51.21)	1138(54.24)	1092(52.02)	1028(49.05)	0.009
Vasopressin use, n (%)	3088(36.80)	612(29.16)	683(32.55)	798(38.02)	995(47.47)	< 0.001
Severity scores						
LODS, mean (SD)	5.24(3.69)	4.51(3.36)	4.77(3.43)	5.36(3.60)	6.30(4.04)	< 0.001
SAPSII, mean (SD)	36.41(14.56)	34.87(13.25)	35.72(13.91)	36.45(14.01)	38.58(16.60)	< 0.001
SOFA, mean (SD)	5.58(4.28)	4.83(3.92)	4.98(3.91)	5.57(4.15)	6.96(4.76)	< 0.001
OASIS, mean (SD)	33.72(9.77)	32.17(9.09)	33.00(9.28)	33.95(9.59)	35.77(10.69)	< 0.001
SIRS, mean (SD)	2.57(0.99)	2.35(1.01)	2.49(1.00)	2.64(0.95)	2.79(0.97)	0.015
APSIII, mean (SD)	52.04(27.54)	46.85(24.87)	48.57(25.80)	51.83(26.48)	60.94(30.52)	< 0.001
Outcome						
Follow-up death, n (%)	3010(35.87)	743(35.40)	778(37.08)	727(34.64)	762(36.35)	0.369
Hospital death, n (%)	1225(14.60)	248(11.82)	298(14.25)	298(14.20)	380(18.13)	< 0.001
1-year death, n (%)	2477(29.52)	606(28.87)	648(30.89)	588(28.01)	635(30.30)	0.157
LOS, days, mean (SD)	15.75(16.74)	13.22(13.76)	14.25(14.18)	16.69(17.74)	18.83(19.95)	< 0.001
ICU-LOS, days, mean (SD)	7.16(9.39)	5.29(7.19)	6.24(8.16)	7.42(9.15)	9.68(11.83)	< 0.001
Invasive ventilation, n (%)	3943(46.99)	717(34.16)	885(42.18)	1077(51.31)	1264(60.31)	< 0.001

TyG index: Q1 (4.02–8.48), Q2 (8.48–8.92), Q3 (8.92–9.43), Q4 (9.43–14.18)

TyG index triglyceride glucose index, SBP systolic blood pressure, DBP diastolic blood pressure, HR heart rate, MI myocardial infarction, CHF congestive heart failure, COPD chronic obstructive pulmonary disease, WBC white blood cell, RBC red blood cell, TG triglyceride, LODS logistic organ dysfunction system, SAPSII simplified acute physiological score II, SOFA sequential organ failure assessment, OASIS oxford acute severity of illness score, SIRS systemic inflammatory response syndrome, APSIII acute physiology score III, LOS length of stay, ICU intensive care unit

^a^Sepsis clinical criteria from The Third International Consensus Definitions for Sepsis and Septic Shock (Sepsis-3) [20]

Based on the number of TyG index measurements, the patients were further divided into 2 groups (Table 2). Before propensity-score matching, gender, weight, ethnicity vital signs, medical history, severity scores, part of comorbidities and laboratory parameters were different between the two groups. After matching, the imbalance was significantly reduced, and the baseline variables were basically comparable between the two groups.

**Table 2:** Baseline characteristics between two groups before and after PSM

Categories	Original Cohort		*P-*value	Matched Cohort		*P-*value
	TyG was measured 1 time during hospital stay (N = 6666)	TyG was measured ≥ 2 times during hospital stay (N = 1726)		TyG was measured 1 time during hospital stay (N = 1417)	TyG was measured ≥ 2 times during hospital stay (N = 1417)	
TyG index, mean (SD)	8.92(0.70)	9.43(1.03)	< 0.001	9.21(0.84)	9.29(0.92)	0.021
TyGVR, mean (SD)	NA	− 0.0006(0.081)	NA	NA	0.0018(0.078)	NA
Demographic						
Age, years, mean (SD)	65.92(16.01)	58.75(16.61)	0.052	60.31(16.64)	60.06(16.49)	0.734
Male, n (%)	3764(56.47)	1037(60.08)	0.007	856(60.41)	841(59.35)	0.592
Emergency, n (%)	5321(79.82)	1379(79.90)	0.973	1147(80.95)	1140(80.45)	0.775
Ethnicity, n (%)			0.024			0.471
White	4300(64.51)	1040(60.25)		904(63.80)	865(61.04)	
Black	590(8.85)	172(9.97)		117(8.26)	143(10.09)	
Asian	180(2.70)	52(3.01)		37(2.61)	42(2.96)	
Hispanic/Latino	205(3.08)	65(3.77)		46(3.25)	54(3.81)	
Other	322(4.83)	80(4.63)		72(5.08)	68(4.80)	
Unknown	1069(16.04)	317(18.37)		241(17.01)	245(17.29)	
Obesity, n (%)	650(9.75)	233(13.50)	< 0.001	172(12.14)	183(12.91)	0.532
Weight, Kg, mean (SD)	82.27(23.76)	87.24(28.79)	< 0.001	86.20(26.81)	86.08(27.72)	0.210
Vital signs						
SBP, mmHg, mean (SD)	123.26(18.41)	117.93(16.87)	< 0.001	118.16(17.00)	118.29(17.19)	0.677
DBP, mmHg, mean (SD)	66.19(12.13)	65.13(11.47)	0.004	64.63(11.43)	64.94(11.57)	0.641
Respiratory rate, bmp, mean (SD)	19.35(3.77)	21.02(4.62)	< 0.001	20.69(4.52)	20.71(4.51)	0.939
HR, bmp, mean (SD)	83.97(16.30)	91.85(17.93)	< 0.001	91.08(18.21)	90.78(17.81)	0.406
Comorbidities						
Diabetes mellitus, n (%)	1986(29.79)	462(26.77)	0.014	365(25.76)	380(26.82)	0.522
Hypertension, n (%)	3782(56.74)	788(45.65)	< 0.001	665(46.93)	661(46.65)	0.910
MI, n (%)	1577(23.66)	230(13.33)	< 0.001	183(12.91)	202(14.26)	0.324
Respiratory failure, n (%)	1619(24.29)	922(53.42)	< 0.001	717(50.60)	704(49.68)	0.652
CHF, n (%)	1774(26.61)	378(21.90)	< 0.001	319(22.51)	323(22.79)	0.893
Cerebrovascular disease, n (%)	2591(38.87)	339(19.64)	< 0.001	285(20.11)	305(21.52)	0.379
Anemia, n (%)	1191(17.87)	437(25.32)	< 0.001	338(23.85)	355(25.05)	0.484
COPD, n (%)	1391(20.87)	434(25.14)	< 0.001	349(24.63)	346(24.42)	0.930
Chronic renal disease, n (%)	1196(17.94)	257(14.89)	0.003	223(15.74)	224(15.81)	1.000
Rheumatic disease, n (%)	221(3.32)	65(3.77)	0.372	49(3.46)	52(3.67)	0.840
Peripheral vascular disease, n (%)	646(9.69)	166(9.62)	0.964	134(9.46)	135(9.53)	1.000
Liver disease, n (%)	870(13.05)	390(22.60)	< 0.001	345(24.35)	319(22.51)	0.268
Dementia, n (%)	261(3.92)	32(1.85)	< 0.001	32(2.26)	27(1.91)	0.599
Sepsis3.0, n (%)	3027(45.41)	1284(74.39)	< 0.001	1076(75.94)	1035(73.04)	0.085
Laboratory parameters						
WBC, K/µL, mean (SD)	10.73(6.55)	12.09(7.50)	< 0.001	11.84(5.57)	11.96(7.71)	0.842
RBC, m/µL, mean (SD)	3.66(0.70)	3.28(0.62)	< 0.001	3.30(0.63)	3.33(0.63)	0.732
Platelet, K/µL, mean (SD)	227.45(101.08)	239.60(127.53)	< 0.001	234.89(129.19)	238.81(126.09)	0.361
Hemoglobin, g/dL, mean (SD)	10.97(2.03)	9.76(1.74)	< 0.001	9.85(1.74)	9.92(1.79)	0.289
Serum Sodium, mEq/L, mean (SD)	139.15(3.80)	139.22(3.81)	0.959	139.19(4.05)	139.24(3.81)	0.741
Serum Potassium, mEq/L, mean (SD)	4.08(0.36)	4.10(0.32)	< 0.001	4.09(0.38)	4.10(0.32)	0.196
Serum Calcium, mg/dL, mean (SD)	8.60(0.59)	8.38(0.57)	0.252	8.39(0.61)	8.39(0.58)	0.879
Scr, mg/dL, mean (SD)	1.28(1.17)	1.41(1.20)	0.130	1.38(1.13)	1.38(1.19)	0.179
Blood urea nitrogen, mg/dL, mean (SD)	24.50(16.99)	29.69(19.97)	< 0.001	29.40(20.60)	29.06(19.73)	0.942
Glucose, mg/dL, mean (SD)	136.43(62.01)	150.50(93.42)	< 0.001	147.32(79.30)	146.54(79.47)	0.937
TG, mmol/L, mean (SD)	143.28(149.35)	267.47(348.38)	< 0.001	200.82(266.30)	221.09(229.12)	< 0.001
Medical history						
Antihypertensive agents, n (%)	5425(81.38)	1476(85.52)	< 0.001	1202(84.83)	1202(84.83)	1.000
Hypoglycemic agents, n (%)	4661(69.92)	1286(74.51)	< 0.001	1056(74.52)	1041(73.47)	0.549
Lipid-lowering agents, n (%)	3710(55.66)	623(36.10)	< 0.001	516(36.41)	535(37.76)	0.484
Vasopressin use, n (%)	2129(31.94)	959(55.56)	< 0.001	781(55.12)	753(53.14)	0.309
Severity scores						
LODS, mean (SD)	4.75(3.43)	7.10(4.01)	< 0.001	7.08(3.95)	6.83(3.93)	0.859
SAPSII, mean (SD)	35.38(14.00)	40.38(15.92)	< 0.001	40.96(16.21)	40.12(15.63)	0.174
SOFA, mean (SD)	5.03(3.97)	7.72(4.75)	< 0.001	7.81(4.68)	7.44(4.66)	0.855
OASIS, mean (SD)	32.72(9.40)	37.59(10.21)	< 0.001	37.81(9.97)	37.38(10.15)	0.496
SIRS, mean (SD)	2.48(1.00)	2.90(0.90)	< 0.001	2.88(0.93)	2.87(0.91)	0.482
APSIII, mean (SD)	48.29(25.47)	66.53(30.30)	< 0.001	65.91(30.60)	64.19(29.58)	0.201
Outcome						
Follow-up death, n (%)	2266(33.99)	744(43.11)	< 0.001	613(43.26)	607(42.84)	0.850
Hospital death, n (%)	851(12.77)	374(21.67)	< 0.001	306(21.59)	296(20.89)	0.679
1-year death, n (%)	1841(27.62)	636(36.85)	< 0.001	530(37.40)	511(36.06)	0.483
LOS, days, mean (SD)	12.85(12.96)	26.94(23.59)	< 0.001	18.52(17.32)	25.56(22.92)	< 0.001
ICU-LOS, days, mean (SD)	5.66(7.21)	12.96(13.63)	< 0.001	9.43(10.19)	11.52(12.46)	< 0.001
Invasive ventilation, n (%)	2743(41.15)	1200(69.52)	< 0.001	958(67.61)	966(68.17)	0.778

TyG index triglyceride glucose index, TyGVR triglyceride glucose index variability ratio, SD standard deviation, PSM propensity score match, NA not available, SBP systolic blood pressure, DBP,diastolic blood pressure, HR heart rate, MI myocardial infarction, CHF congestive heart failure, COPD chronic obstructive pulmonary disease, WBC white blood cell, RBC red blood cell, TG triglyceride, LODS logistic organ dysfunction system, SAPSII simplified acute physiological score II, SOFA sequential organ failure assessment, OASIS oxford acute severity of illness score, SIRS systemic inflammatory response syndrome, APSIII acute physiology score III, LOS length of stay, ICU intensive care unit

^a^Sepsis clinical criteria from The Third International Consensus Definitions for Sepsis and Septic Shock (Sepsis-3) [20]

### Association of TyG index and TyGVR with all-cause mortality

**Fig.2:**
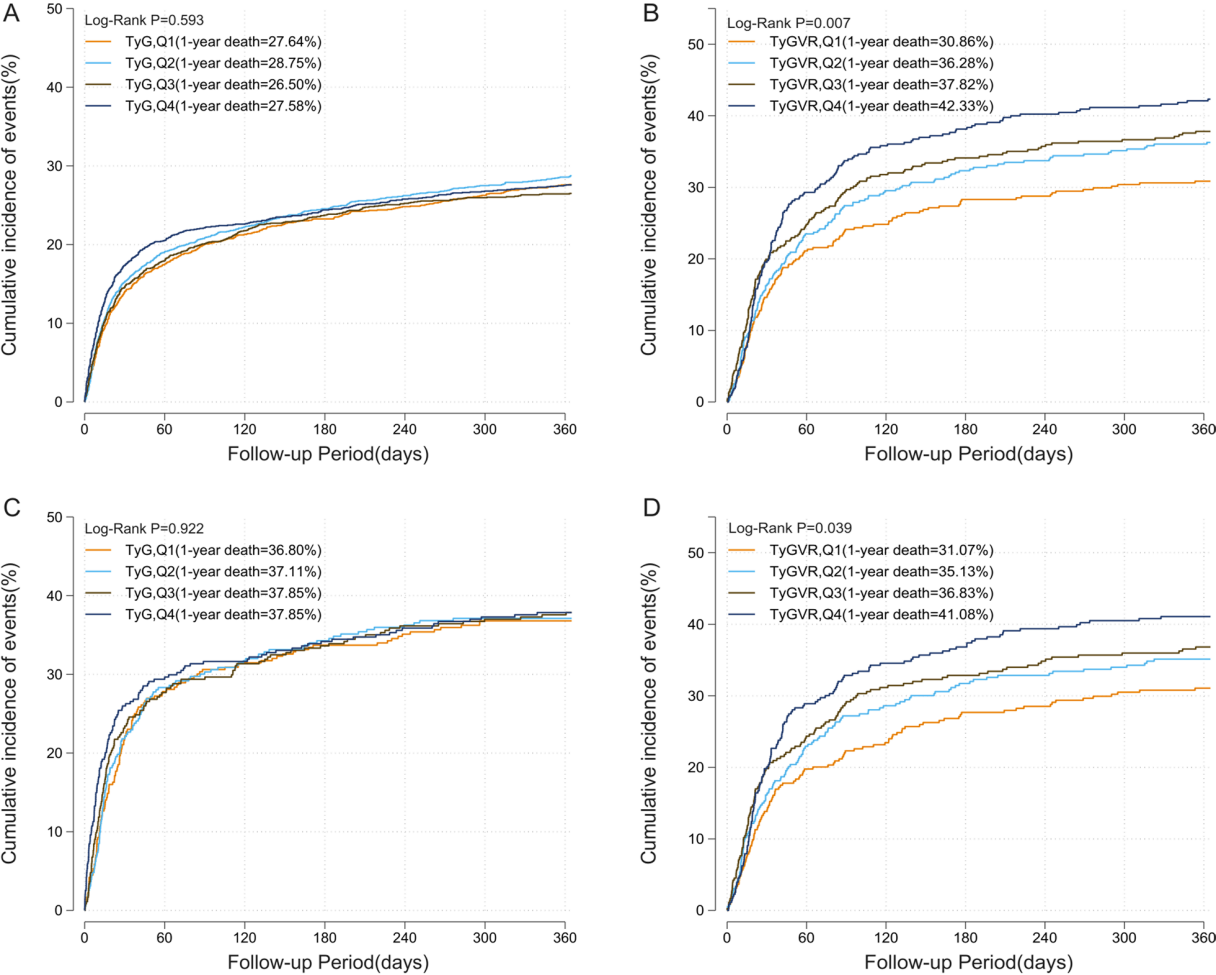
Kaplan–Meier survival analysis curves for all-cause mortality. Before PSM, TyG index: Q1 (4.02–8.48), Q2 (8.48–8.92), Q3 (8.92–9.43), Q4 (9.43–14.18). TyGVR: Q1 (-0.2995–-0.0401), Q2(-0.0401–0.0001), Q3(0.0001–0.0408), Q4(0.0408–0.4077). After PSM, TyG index: (7.00–8.62), Q2 (8.62–9.11), Q3 (9.11–9.68), Q4 (9.68–13.66). TyGVR: Q1 (-0.2995–-0.0401), Q2(-0.0401–0.0001), Q3(0.0001–0.0408), Q4(0.0408–0.4077). Kaplan–Meier curves showing cumulative probability of 1-year death according to (**A**), quartile of TyG index before PSM (**B**), quartile of TyGVR before PSM (**C**), quartile of TyG index after PSM (**D**), quartile of TyGVR after PSM.TyG index triglyceride glucose index, TyGVR triglyceride glucose index variability ratio, PSM propensity score match

**Fig.3:**
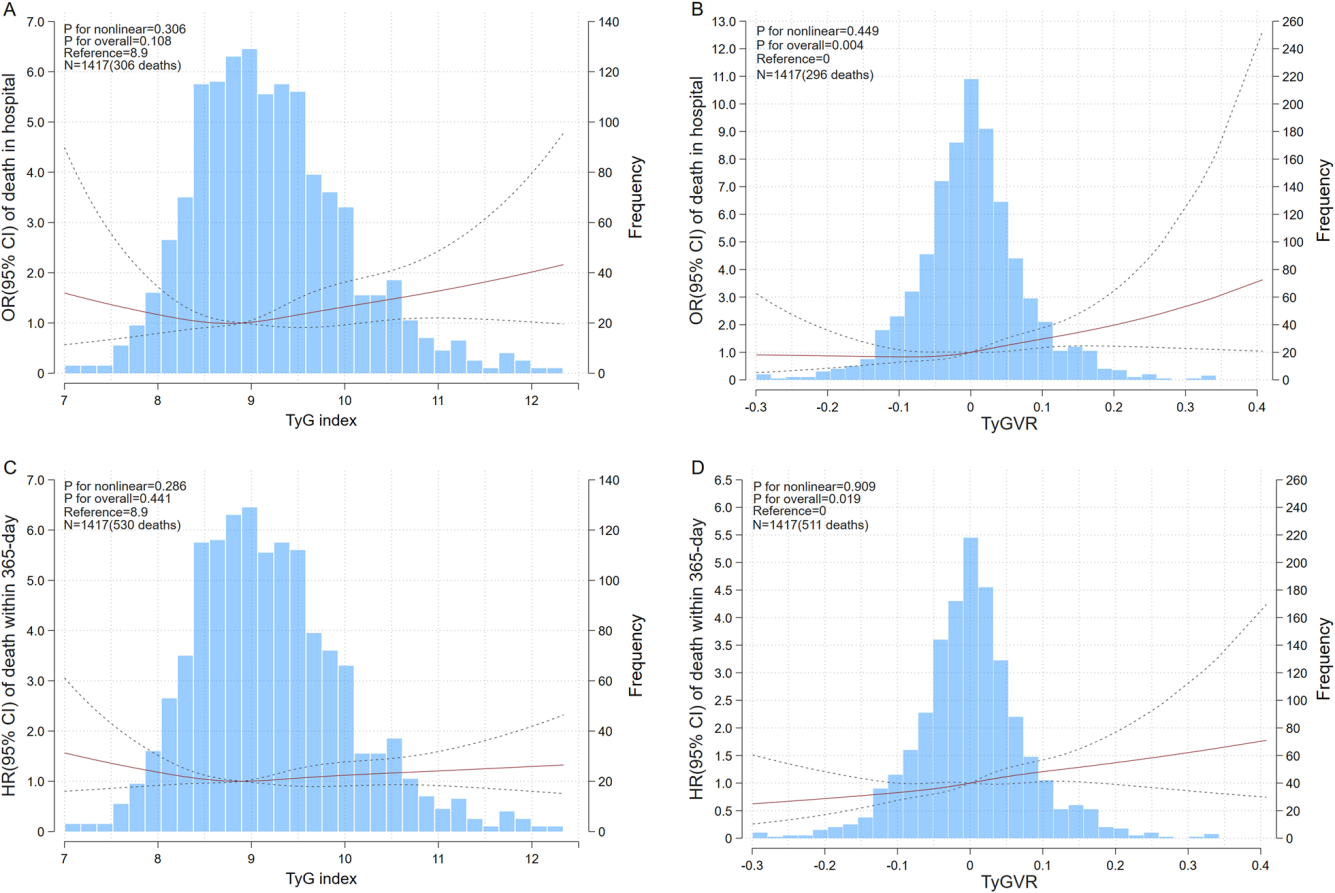
Potential nonlinear for the levels of TyG index and TyGVR with in-hospital death and 1-year death measured by restricted cubic spline regression with 4 knots located at the 5^th^, 35^th^, 65^th^ and 95^th^ percentiles. The red and dotted lines represent the estimated HR or OR and the 95% CI, respectively. TyG index 8.9 was selected as the reference level. 0 was selected as the TyGVR reference level. (**A**), TyG and in-hospital death in matched cohort (**B**), TyGVR and in-hospital death in matched cohort (**C**), TyG and 1-year death in matched cohort (**D**), TyGVR and 1-year death in matched cohort. HR hazard ratio, OR odds ratio, CI confidence interval, TyG index triglyceride glucose index, TyGVR triglyceride glucose index variability ratio

Kaplan–Meier curves for assessing 1-year all-cause mortality in critical patients on basis of TyG index and TyGVR were presented in Fig. 2. In the original cohort, patients with higher TyGVR had significantly higher 1-year all-cause mortality (Q1: 30.86% vs. Q2: 36.28% vs. Q3: 37.82% vs. Q4: 42.33%, log-rank P = 0.007, Fig. 2B) than those lower TyGVR, while no difference was found among TyG index groups (Q1: 27.64% vs. Q2: 28.75% vs. Q3: 26.50% vs. Q4: 27.58%, log-rank P = 0.593, Fig. 2A). Similarly, in the matched cohort, the cumulative incidence of all-cause death increased with higher quartile of the TyGVR (Fig. 2D). But there were no differences in TyG index (Fig. 2C). Restricted cubic spline analysis after PSM demonstrated a nearly linear association between TyGVR and the risk of in-hospital (P for non-linear = 0.449, P for overall = 0.004, Fig. 3B) and 1-year all-cause mortality (P for non-linear = 0.909, P for overall = 0.019, Fig. 3D), while no significant association was found between TyG index and all-cause mortality. (Fig. 3A and Fig. 3C).

**Fig.4:**
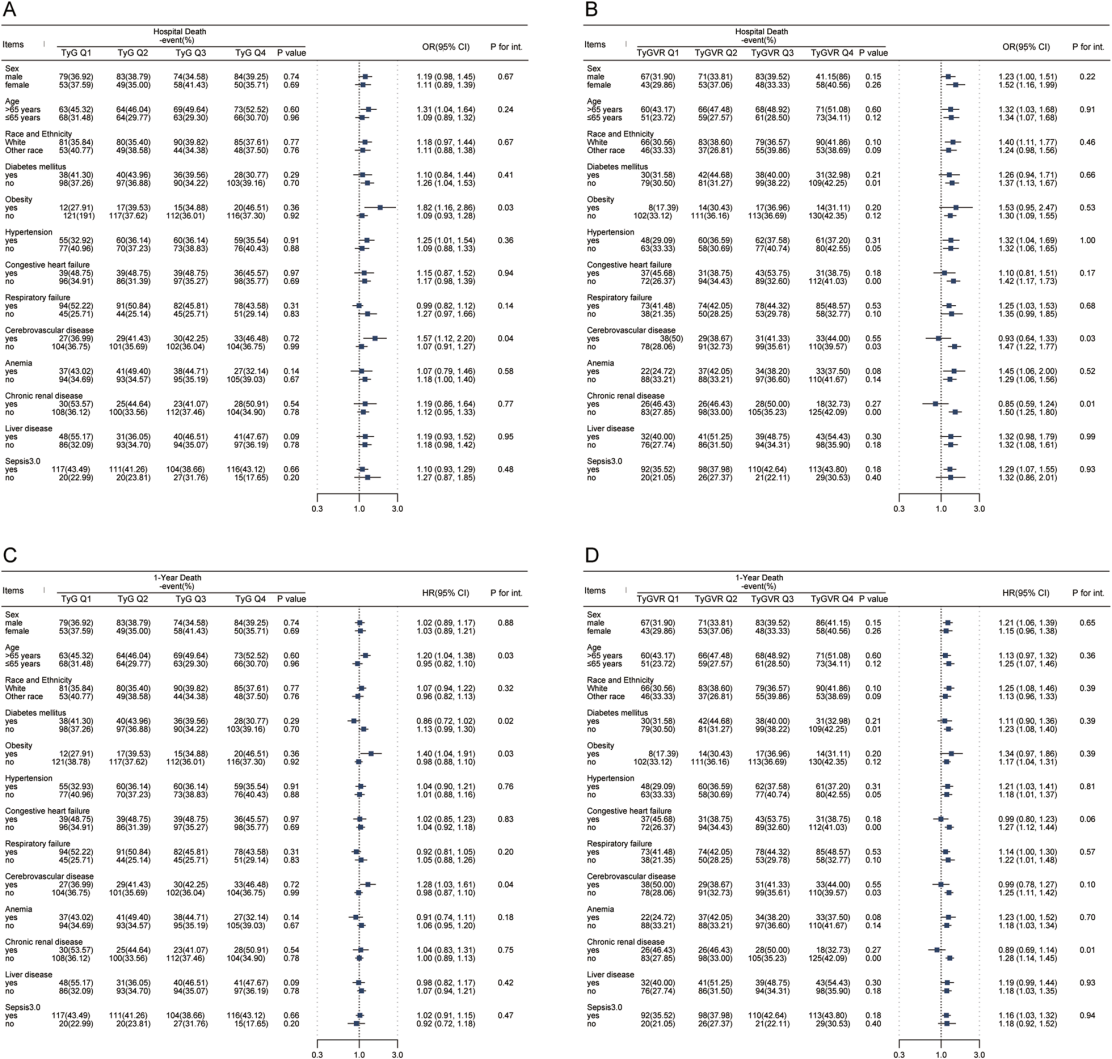
Subgroup analyses for the association of TyG index and TyGVR with in-hospital dearh and 1-year death. (**A**), TyG and in-hospital death in matched cohort (**B**), TyGVR and in-hospital death in matched cohort (**C**), TyG and 1-year death in matched cohort (**D**), TyGVR and 1-year death in matched cohort. HR hazard ratio, OR odds ratio, CI confidence interval, TyG index triglyceride glucose index, TyGVR triglyceride glucose index variability ratio

Figure 4 shows the results of stratified analyses. Overall, the relationship between TyG index and TyGVR with all-cause mortality was consistent across most sub-populations. Nevertheless, for TyG index, a higher prevalence of increased in-hospital mortality (Fig. 4A) in critical patients with obesity [OR (95% CI) 1.82 (1.16, 2.86), P for interaction = 0.03], cerebrovascular disease [OR (95% CI) 1.57 (1.12, 2.20), P for interaction = 0.04], whereas, for 1-year all-cause mortality(Fig. 4C), those age > 65 years [HR (95% CI) 1.20 (1.04, 1.38), P for interaction = 0.03] and those without diabetes mellitus [HR (95% CI) 1.13 (0.99, 1.30), P for interaction = 0.03] showed higher prevalence. On the other hand, TyGVR was consistently associated with the risk of in-hospital (Fig. 4B) and 1-year all-cause mortality (Fig. 4D) among different subgroups, except for cerebrovascular disease (interaction P for in-hospital mortality = 0.03, for 1-year all-cause mortality = 0.10).

### Incremental effect of TyG index and TyGVR on predictive value for all-cause mortality

ROC and AUC analysis were performed to evaluate the value of TyG index and TyGVR combined with the existing severity of illness scores for in-hospital and 1-year all-cause mortality. As shown in Additional file 2: Fig.S1, the AUC for in-hospital mortality significantly improved with the addition of TyG index (Additional file 2: Fig.S1A) and TyGVR (Additional file 2: Fig.S1B) to conventional severity of illness scores. Similar results were observed for 1-year all-cause mortality (Additional file 2: Fig. S1C, D, all P < 0.01).

### Association of TyG and TyGVR with all-cause mortality

Linear regression was used to examine the correlation between TyG index, TyGVR, and LOS. The results showed that neither TyG nor TyGVR had a significant association with LOS in patients who survived the hospital stay (TyG model:β = -0.92, P = 0.09, TyGVR model:β = 7.14, P = 0.36) or those who survived the ICU stay (TyG model:β = 0.45, P = 0.16, TyGVR model:β = 3.41, P = 0.43, Additional file 1: Table S1).

## Discussion

To our knowledge, this study is the first to demonstrate that higher TyGVR levels are associated with increased long-term all-cause mortality risk in critically ill patients, even after adjusting for potential confounding factors. Specifically, our data suggest that TyGVR confers a greater long-term mortality risk than TyG. Additionally, both TyG and TyGVR are strong predictors of in-hospital mortality in ICU patients. Furthermore, the addition of TyGVR, in comparison with TyG, to baseline severity scores make an incremental effect on the predictive value for all-cause mortality. Similarly, significant but relatively weaker abilities for TyG on mortality prediction were obtained. Notably, the risk of hospital and 1-year mortality increases linearly with increasing TyGVR levels, but this trend is absent for TyG. Most importantly, our study addresses the gap in knowledge regarding the dynamic changes of TyG, a simple biomarker that may reflect the state of illness, which can aid in optimizing in-hospital and long-term risk stratification of mortality. This information is essential for better clinical management and reducing future mortality events.

The metabolic response to critical illness is a part of the adaptive response and involves multiple organ systems, whereby energy reserves are directed to where they are most needed. Several mechanisms are elicited to augment the provision of energy substrates to the vital tissues, such as the activation of the sympathetic nervous system, secretion of pituitary hormones, and peripheral resistance to the effects of anabolic factors [23]. The oxidation of carbohydrates is globally more increased during the early phase than the oxidation of lipids and proteins [24]. Later on, decreased glucose utilization, increased fat turnover, and loss of muscle and visceral (organ) protein mass with wasting occur [25]. The liver produces large amounts of glucose, from glycogenesis and neoglucogenesis. Glucose will be mainly used by non-insulin-dependent organs, while lipolysis will occur in fat tissue and proteolysis in muscles. Free fatty acid released by lipolysis are highly susceptible to peroxidation by reactive oxygen species massively released after stress-induced mitochondrial dysfunction [26]. Glycerol released from lipolysis will be regenerated by the liver into glucose [27, 28]. Muscular proteolysis will release amino acids that will be recycled into glucose (mainly alanine and glutamine) or degraded into urea or ammonium [23]. Lactate generated in hypoxic areas will be used by the liver to generate glucose by the Cori cycle [23]. The final common pathway of the metabolic response to critical illness implies an uncontrolled catabolism and the development of a resistance to anabolic signals, including insulin, in order to reset the hierarchy of the delivery of energy substrates to prioritize vital tissues over the insulin-dependent organs [29, 30].

IR evaluated by TyG index has been proven to be a high correlation with hyperinsulinaemic–euglycaemic clamp [31]. In light of low-cost routine biochemical detection and good application prospect, TyG index is widely used as a marker of IR in various clinical settings. Numerous prior studies [32, 33] have mainly focused on the relationship between baseline TyG index and the prevalence and prognosis of cardiovascular or cerebrovascular disease. A meta-analysis from Yan et al. [32] found that there is a potential linear dose-response relationship between baseline TyG index and cerebrovascular disease. Tao et al. reported a review [33] that TyG index can be used as a reliable and convenient surrogate for IR, which can be optimized for risk stratification as well as outcome prediction for cardiovascular disease. However, current data on critically ill patients are limited. Zhai et al. conducted a multicenter study on critically ill stroke patients [19], which suggested that the TyG index has the potential to predict hospital and ICU mortality in this patient population. Another study by Liao et al. [16] enrolled unselected ICU adult patients found that TyG index was an independent predictor of hospitalization and ICU mortality in critically ill patients. Additionally, a recent study by Zhang et al. [34] demonstrates that the baseline TyG index was significantly linearly correlated with the risk of all-cause mortality in critically ill patients with (coronary heart disease) CHD [34]. However, ICU patients experience dynamic and progressive disturbances, and the development of acute diseases such as sepsis, shock, or trauma can lead to stress hyperglycemia. This can potentially bias the diagnostic or predictive value of the TyG index [33]. Most prior studies use the baseline TyG index as a biomarker to predict outcomes. This may be less robust. Research from Zauner et al. [6] has shown that IR was related to the severity of their condition in ICU patients, regardless of their admission diagnoses [6]. Thus, it is essential to evaluate the dynamic changes in the TyG index in the state of an illness progression during ICU stay. A prospective cohort from Kailuan study [35] revealed cumulative TyG index (defined as the summation of average TyG index for each pair of consecutive evaluations multiplied by the time between these two consecutive visits in years) was associated with an increased risk of (cardiovascular disease) CVD. To the best of our knowledge, no cohort study has explored the relationship between TyG index and critically ill patients by repeated measurements analysis. Our finding makes the study be great agreement and complement to previous literature.

Another finding from subgroup indicates that higher TyGVR seems to significantly increase the risk of all-cause mortality in critical patients without (chronic kidney disease) CKD. This phenomenon seems to contradict previous studies. A prospective study from Zhao et al. [36] found that an elevated TyG index was significantly associated with a higher risk of nephric microvascular damage. Sikandar et al. [37] reported that the TyG index demonstrated a positive linear correlation with urine albumin to creatinine ratio. In contrast, Zhang et al. [34] did not find any association between the TyG index and in-hospital all-cause mortality in participants with CKD at baseline. This outcome could not be fully explained by reverse causality [38] and may partly attribute to another two reasons: firstly, IR plays a more important role in metabolic disease-induced CKD by causing hyperglycemia and later low-grade inflammation and fibrosis. Compare with the general population [39], TyG index might represent different pathophysiological states in critically ill individuals, secondly, monitoring TyG index dynamic change during hospital stay may more accurately reflect the development of kidney disease. Just recording the baseline TyG index at admission is not enough.

## Study strengths and limitations

The most strength of the current study is the repeated assessment of TyG index during hospital stay has important implications for critically ill patients. The TyGVR might help stratify critically ill individuals at high risk for all-cause mortality, whether during hospital stay or long-term follow-up. Further research is needed to validate the relationship between the mean changes of TyG index in critically ill patients during hospital stay across various chronic diseases and mortality.

As the nature of single-center retrospective study, limited sample size and data bias could be inevitable despite vigorous statistical correction being performed. Additionally, we could provide only the association between TyGVR and mortality rather than causality. Furthermore, although we adjusted for other relevant confounders including obesity, we did not record dietary habits and energy intake that might dramatically affect TG levels. Finally, the data of death is derived from hospital records and state records. The etiology of death, particularly out-of-hospital death, remains unclear. A well-designed prospective study should be conducted to evaluate the causality between TyGVR and mortality in the future.

## Conclusions

In summary, IR dynamic change, repeatedly assessed by accessible and reliable TyG index, during hospital stay was positively and independently associated with an increased risk for all-cause mortality in individuals with critical illnesses. Moreover, the dynamic change of TyG index may provide more valuable information than the baseline TyG index in identifying patients at high-risk all-cause mortality. Future work should focus on the clinical implications of assessment of TyG index variability across different clinical states.

## Supplementary Information

**Additional file 1: Table S1.** Association of TyG and TyGVR with length of stay

**Additional file 2: Figure S1.** Area under ROC curve of TyG index and TyGVR combined with various scores for in-hospital mortality and 1-year mortality.

## Data Availability

The datasets generated and analyzed during the current study are available from the corresponding author on reasonable request.

## Abbreviations

TyG: Triglyceride-glucose
ICU: Intensive care unit
MIMIC-IV: Medical Information Mart for Intensive Care IV
TyGVR: Triglyceride glucose index variability ratio
AUC: Area under the curve
APACHE: Acute physiology and chronic health evaluation
SAPS II: Simplified acute physiological score II
IR: Insulin resistance
TG: Triglyceride
FBG: Fasting blood glucose
COVID-19: Coronavirus disease 2019
ICD: International Classification of Diseases
STROBE: Strengthening the Reporting of Observational Studies in Epidemiology
LOS: Length of stay
SD: Standard deviation
IQR: Interquartile range
PSM: Propensity score match
ASD: Absolute standardized difference
HR: Hazard ratio
OR: Odds ratio
CI: Confidence interval
ROC: Receiver operating characteristic
WBC: White blood cell
CHD: Coronary heart disease
CVD: Cardiovascular disease
CKD: Chronic kidney disease
