# Supplementary material for: Association of dynamic change of triglyceride-glucose index during hospital stay with all-cause mortality in critically ill patients: a retrospective cohort study from MIMIC IV2.0
Source: Cardiovasc Diabetol. 2023 Jun 17;22:142. doi: 10.1186/s12933-023-01874-9 (PMC10276426; doi:10.1186/s12933-023-01874-9)
Supplement: Supplementary file 1 — Additional file 1: Table S1. Association of TyG and TyGVR with length of stay [file 12933_2023_1874_MOESM1_ESM.docx]

Table S1 Association of TyG and TyGVR with length of stay (LOS)

|  | Coef. | S.E. | t-value | *P*-value |
| --- | --- | --- | --- | --- |
| LOS hospital |  |  |  |  |
| TyG index model | -0.92 | 0.55 | -1.68 | 0.09 |
| TyGVR model | 7.14 | 7.86 | 0.91 | 0.36 |
| LOS ICU |  |  |  |  |
| TyG index model | 0.45 | 0.32 | 1.40 | 0.16 |
| TyGVR model | 3.41 | 4.27 | 0.80 | 0.43 |

Model: all model was unadjusted.

TyG index triglyceride glucose index, TyGVR triglyceride glucose index variability ratio
