# Supplementary material for: Association of dynamic change of triglyceride-glucose index during hospital stay with all-cause mortality in critically ill patients: a retrospective cohort study from MIMIC IV2.0
Source: Cardiovasc Diabetol. 2023 Jun 17;22:142. doi: 10.1186/s12933-023-01874-9 (PMC10276426; doi:10.1186/s12933-023-01874-9)
Supplement: Supplementary file 2 — Additional file 2: Figure S1. Area under ROC curve of TyG index and TyGVR combined with various scores for in-hospital mortality and 1-year mortality. [file 12933_2023_1874_MOESM2_ESM.docx]

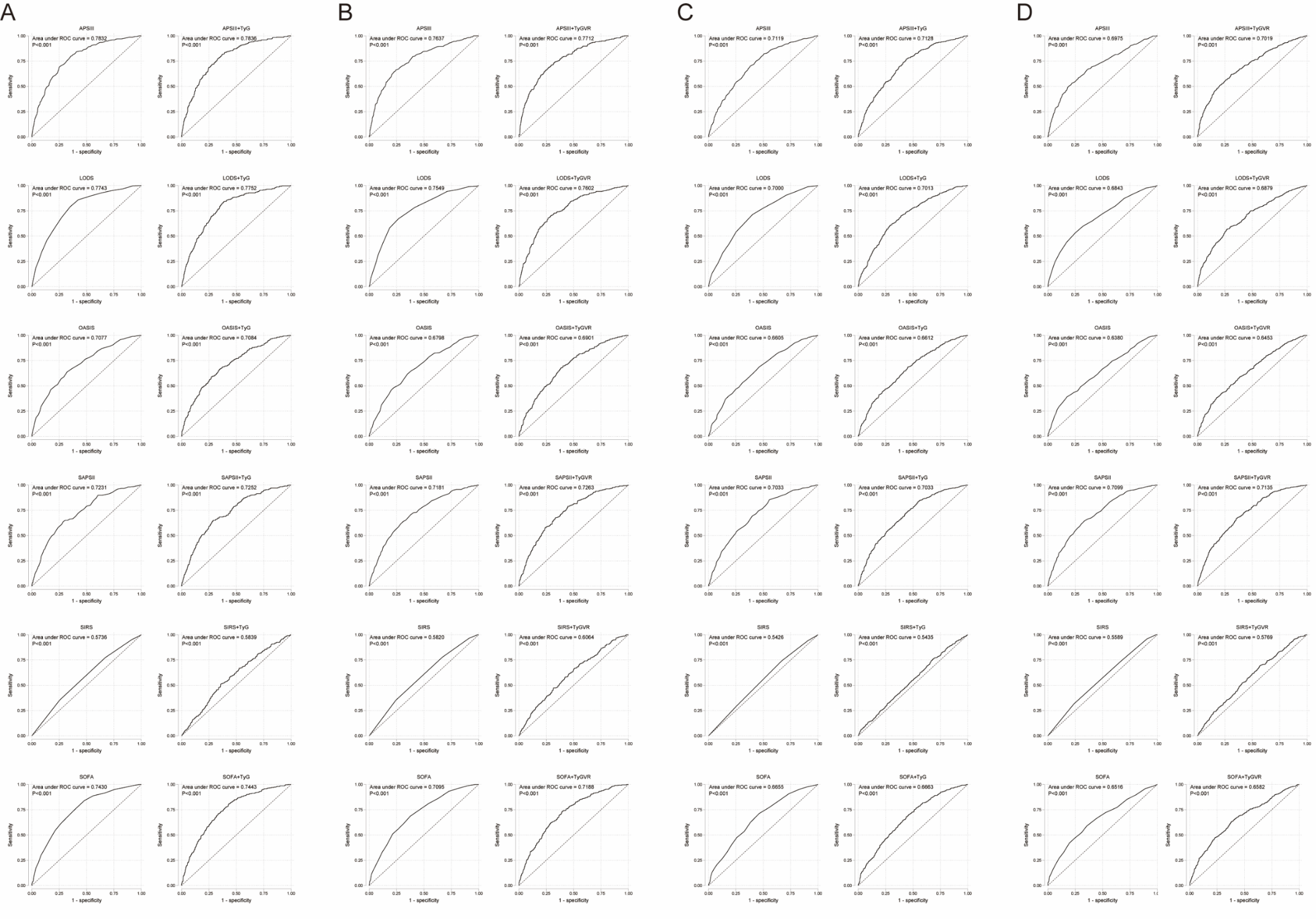


| Figure S1 Area under ROC curve of TyG index and TyGVR combined with various scores for in-hospital mortality and 1-year mortality. (**A**), Discrimination of TyG index combined various scores for hospital death, (**B**), Discrimination of TygVR combined various scores for hospital death, (**C**), Discrimination of TyG index combined various scores for 1-year death, (**D**), Discrimination of TyGVR combined various scores for 1-year death, TyG index triglyceride glucose index, TyGVR triglyceride glucose index variability ratio, APSIII acute physiology score III, LODS logistic organ dysfunction system, OASIS oxford acute severity of illness score, SAPSII simplified acute physiological score II, SIRS systemic inflammatory response syndrome, SOFA sequential organ failure assessment. |
| --- |
